# Reducing hospital stay for colorectal surgery in ERAS setting by means of perioperative patient education of expected day of discharge

**DOI:** 10.1007/s00384-021-03948-0

**Published:** 2021-05-11

**Authors:** Thaís T. T. Tweed, Carmen Woortman, Stan Tummers, Maikel J. A. M. Bakens, James van Bastelaar, Jan H. M. B. Stoot

**Affiliations:** Department of Surgery, Gastrointestinal Surgery, Zuyderland Medical Centre, Dr. H. van der Hoffplein 1, 6162BG Sittard-Geleen, The Netherlands

**Keywords:** Perioperative education, Colorectal cancer, Gastrointestinal, Enhanced recovery after surgery

## Abstract

**Purpose:**

Despite the enhanced recovery after surgery (ERAS) protocol, length of stay (LOS) after colorectal surgery varies considerably. The majority of longer admissions is often not medically necessary. We aimed to investigate possible reduction of LOS by perioperative education with an expected discharge date (EDD).

**Methods:**

This single-centre retrospective study included 578 patients who underwent surgery for colorectal cancer in 2016 with standard care (ERAS) and in 2018 with the addition of EDD education program (ERAS+). A comparison was made of a 1-year period prior to and following the implementation of EDD. The EDD was discussed at the outpatient clinic, preoperatively and during admission (with both the patient and family members daily). Standard EDD varied between 3 and 5 days depending on the resection type. Primary outcome was LOS; secondary outcomes were readmission, serious complications and 90-day mortality.

**Results:**

Patients in ERAS+ (*n* = 242) had a shorter median LOS (4.0 vs. 5.0, *p* < 0.001) compared to patients in the regular ERAS group (*n* = 336). Fewer patients of ERAS+ experienced postoperative complications (71 (29.3%) vs. 198 (58.9%), *p* < 0.001). No difference was found in the number of readmissions (23 (9.5%) vs. 34 (10.1%), *p* = 0.807), reinterventions (25 (10.3%) vs. 30 (8.9%), *p* = 0.571) or mortality (5 (2.1%) vs. 9 (2.7%), *p* = 0.261) between the two groups.

**Conclusion:**

It is possible to reduce LOS within the ERAS program, by better perioperative education and expectation management of patients with use of an EDD. This program ensures better understanding, faster discharge and lower costs for the hospital without added risk of readmissions or complications.

## Introduction

Since the development and introduction of enhanced recovery after surgery (ERAS) programs, also referred to as fast track perioperative care, the average length of hospital stay (LOS) after colorectal cancer surgery was reduced by 30 to 50% [1, 2]. The principal behind this enhanced recovery lies in optimizing perioperative care through pre-, intra- and postoperative measures [3], focusing not only on optimizing surgical care, but also non-surgical care. Enhanced postoperative recovery is enabled by patient education, early oral feeding, early mobilization, minimally invasive surgery and optimal pain control [4, 5]. In patients undergoing colorectal surgery, the ERAS program has proven to enable earlier functional recovery and a reduction of postoperative morbidity, resulting in shorter length of hospital stay [6, 7].

However, not all patients are discharged earlier after colorectal surgery following the ERAS program. Despite the implementation of the ERAS program, some reports have described that the most common reason for a longer hospital stay is non-medical related [8]. The discharge procedure is largely determined by the ERAS discharge criteria and willingness to go home. Functional recovery is defined as sufficient intake (60% of the required intake), normal mobilization, recovery of bowel function (flatus or stool) and adequate pain control with oral analgesics (VAS score ≤ 3 in rest and ≤ 5 in upon mobilization) [9, 10]. One of the causes of the unnecessary delay was the patients’ unwillingness to return home [11]. Apart from discharge management by attending staff, patients’ psychological factors such as motivation or fear of returning home can also influence the moment of discharge [12]. An efficient education of patients preoperatively has been reported to reduce anxiety effectively [13, 14]. Lack of patient education and expectation management could lead to a larger gap between time-to-readiness of discharge and actual discharge day [15]. In addition, increased risk of complications, longer hospital stays and readmissions have been described as consequences of ineffective education [12]. Research presented a progressive need of patients to obtain more information and to be actively involved in their care process from diagnosis to recovery [16]. This study investigated the perioperative education of expected day of discharge (EDD). The aim of the study was to reduce LOS by engaging patients more actively, with informing EDD preoperatively and during hospital stay by reminding patients and their respected families on a daily basis as well as attending staff.

## Methods

This study is a single-centre retrospective cohort analysis from the Zuyderland Medical Centre. A comparison was made of a 1-year period prior to and following the implementation of EDD, which was called the ERAS+ education program. The new ERAS+ education program (ERAS + EDD) was implemented in 2017. Therefore, patients operated in 2016 (regular ERAS) were compared to patients operated in 2018 (ERAS+) to exclude the transition period and to minimize bias.

### Participants

All patients were included who electively underwent surgical resection for colorectal cancer between the first of January 2016 until the 31st of December 2016, and the first of January 2018 until the 31st of December 2018. No specific exclusion criteria on age, sex or comorbidities were applied. When the EDD was not applied due to unexpected change of surgical procedure and/or complication during surgery, patients from the 2018 cohort were excluded. All patients received usual care following the ERAS protocol including guidance of a dedicated trained ERAS nurse [4]. All patients received an ERAS information booklet which further explained the pre-, per- and postoperative procedures, as part of standard care.

### Standard enhanced recovery after surgery protocol

All patients scheduled to undergo any form of colorectal resection were assigned to a dedicated ERAS nurse, who explained the enhanced recovery after surgery protocol to the patients (in the weeks) prior to surgery. These nurses would hand out an ERAS-information booklet to the patients and were reachable by phone in case patients had more questions concerning their planned admission. If found to be functionally compromised/frail, patients 70 years or older were referred for an in-depth assessment by the geriatrician. Patients with a Short Nutritional Assessment Questionnaire (SNAQ) score of 3 or higher were referred to a dietician for nutritional counselling. During the (first) consultation with the surgeon, patients were once again reminded of the enhanced recovery protocol.

Depending on the time of the scheduled operation, patients were either admitted a day prior to surgery or early on the day of surgery itself. All patients scheduled to undergo a left-sided or rectal resection received bowel preparation with colex enema. Patients who were scheduled to be operated laparoscopically additionally received 10 mg bisacodyl the night before the operation, and 5 mg the morning of the operation. Patients scheduled to undergo right-sided resection were not subjected to bowel preparation. Fasting protocols were as follows: up to 6 h prior to surgery, solid nutrition was allowed. Two hours prior to surgery oral intake was reduced to a clear liquid diet. Carbohydrate loading was achieved by offering patients 2 servings or PreOp carbohydrate drink 1 h prior to surgery.

Routine anaesthesia consisted of a continuous dose of propofol and rocuronium. Doses were based on the age, weight and comorbidities of the patient. In addition to routine anaesthesia, preemptive analgesia with 1 g acetaminophen (paracetamol) was given (orally). Intraoperative fluid administration consisted of 0.5–1.5 L of NaCl throughout surgery, depending on duration and type of surgery. Upon arrival on the operation theatre, patients were covered by warmer blankets and preoperatively warmed with the use of the Bair Hugger forced air patient warming system.

After surgery, patients were encouraged to start early oral intake and were offered an icicle and water on the recovery ward. Postoperative fluid management was adjusted according to patient intake. If patients were nauseated, the anti-emetic protocol was used which consisted of 4 mg dexamethasone intravenously. Postoperative analgesic regimen consisted of 1 g acetaminophen every 6 h and 10 mg morphine subcutaneously if needed. Patients were continuously encouraged to mobilize after surgery. Routine bloodwork was done on postoperative days 3 and 5 to evaluate the inflammatory status of the patient.

### Surgical procedure

Operations were performed by both surgeons and (supervised) surgical residents. The surgical approach (open or laparoscopic) was based on both patient and surgeon preferences. If both options were possible, the choice depended on patient’s preference. Routine D2-lymph node dissection was performed in all cases according to the Dutch Oncologic Guidelines [17]. Continuity was reconstructed based on surgeon’s preference and experience. When possible, reconstruction of the anastomosis was performed intracorporeally. Drains and urinary catheters were not routinely placed postoperatively, only upon indication.

### ERAS+ protocol

The ERAS+ program consisted of the standard ERAS protocol previously described, with the addition of the preoperative education of an EDD. Set discharged dates were determined for each type of colorectal resection (without discrimination of resection approach: open or laparoscopic). Discharge of patients after a right hemicolectomy was set at postoperative day (POD) 3, left hemicolectomy or sigmoidal resection at POD 4 and rectal resections at POD 5. Patients were educated in the outpatient clinic twice preoperatively: first by a dedicated trained ERAS nurse, in a second appointment by the attending surgeon. Prior to hospital admission, a whiteboard was installed in the patient’s room to write down the exact EDD, e.g. May 12th 2018. As soon as the patients arrive on the surgical ward from the recovery ward, they would be reminded of their EDD. The visiting nurses, attending resident and surgeon on the ward, reminded the patient and their family of the EDD on a daily basis.

In order to guarantee safe discharge, the following discharge criteria from the ERAS program were used (16):

- Normal oral intake without nausea and/or vomiting
- Mobilization at preoperative level
- Adequate pain control with oral analgesics
- Passage of flatus

Both the discharge criteria and a matching clinical evaluation had to be met before discharge. If patients did not meet the discharge criteria at the EDD, were insecure about stoma-care, later availability of at home assistance, or other social concerns, discharge was postponed. Discharges to nursing homes were reported as regular discharge.

### Data collection

All demographical, surgical and perioperative outcome data were collected in a prospectively coded database. Data were retrieved from the electronic patient files and double checked with the national Dutch Institute for Clinical Auditing (DICA) database. All postoperative outcomes were gathered from admission up to 90 days after discharge. All procedures performed in this study were in accordance with the ethical standards of the institutional research committee, Medical Ethics Committee of Zuyderland Medical Centre and with the 1964 Helsinki declaration and its later amendments or comparable ethical standards. Ethical approval was sought and granted for this study by Medical Ethics Committee of Zuyderland Medical Centre (study record: METCZ20200091).

The primary outcome was length of stay, determined as the number of days spent in the hospital from first postoperative day to discharge (date of discharge minus date of surgery). Secondary outcomes were number of patients discharged on the EDD, morbidity, readmissions, reinterventions and severe complications according to the Clavien Dindo classification [18] and mortality.

### Statistical analysis

The primary outcome was length of stay. The Student’s *t*-test was used for parametric continuous data, which are presented as mean and standard deviation. For non-parametric data, the Mann-Whitney *U* test was performed and presented as median with interquartile ranges. Categorical data variables were descripted as means with percentages, which were compared with chi-square or Fisher extract test. Multivariate linear regression was used to correct possible confounder and to create the best fitting model. A two-tailed *p*-value of ≤ 0.05 was considered statistically significant. All statistical analyses were performed using the IBM SPSS statistics software program, version 25.0.

## Results

A total of 579 patients were eligible for this study, of which 243 patients were treated in accordance with the ERAS+ protocol, and 336 with regular ERAS care. One eligible patient was excluded after stenting based on an unexpected change in surgical procedure, needing immediate (open) extensive intervention other than previously planned. A total of 242 patients were included in the ERAS+ group, and 336 patients were included in the regular ERAS group. Patient characteristics for both study groups are summarized in Table 1. All but two baseline characteristics were comparable between the two groups. About half of the patients in ERAS+ were males (49.6%), and 61.9% of patients in the regular ERAS group were males (*p* = 0.003). Patients of the ERAS+ group were on average about 2 years older compared to patients of the regular ERAS group (*p* = 0.019). The preoperative status and the Union for International Cancer Control (UICC) TNM Classification of Malignant Tumours stages were comparable between the two study groups.

**Table 1:** Patient characteristics

		ERAS+	n	ERAS	n	*p*-value
Sex						0.003
	Male Female	49.6% 50.4%	120 122	61.9% 38.1%	208 128	
Age	(Median, IQR)	73.8 (64.5–79.4)	242	71.2 (62.8–76.4)	336	0.019
	(Mean, SD)	71.8 (±10.6)		70.1 (±10.2)		
UICC stage						0.056
	Stadium 0	2.5%	6	3.3%	11	
	Stadium IA	8.7%	21	9.5%	32	
	Stadium IB	21.1%	51	14.0%	47	
	Stadium IIA	35.1%	85	35.7%	120	
	Stadium IIB	19.8%	48	18.8%	63	
	Stadium IIIA	7.0%	17	8.6%	29	
	Stadium IIIB	2.1%	5	2.1%	7	
	Stadium IV	3.7%	9	8.0%	27	
CCI						0.235
	0–3	11.6%	28	8.9%	30	
	4–6	55.8%	135	52.1%	175	
	>7	32.6%	79	39.0%	131	
Procedure						0.419
	Open*	13.6%	33	16.1%	54	
	Laparoscopic	86.4%	209	83.9%	282	

Continues variables reported as mean; categorical variables reported as percentages. ERAS describes patients from the 2016 cohort, ERAS+ from the 2018 cohort

*IQR* interquartile range, *SD* Standard deviation, *UICC* the Union for International Cancer Control (UICC) TNM Classification of Malignant Tumours, *CCI* Charlson Comorbidity Index, *Procedure* performed surgical technique

*p*-value key: *Conversions included, 24 cases in both groups

### Primary outcome length of stay

Postoperative length of stay was shorter in the ERAS+ group than in the regular ERAS group as reflected by the median (Table 2). Patients in the ERAS+ group were admitted 1 day less than patients in the ERAS group (median length of stay) (*p* < 0.001).

**Table 2:** Primary and secondary outcomes

	ERAS+	n	ERAS	n	*p*-value
Median length of stay (median, IQR)	4.0 (3.0–6.0)	242	5.0 (4.0–8.0)	336	<0.001
Mean length of stay	5.4 (±4.58)	242	7.2 (±6.90)	336	< 0.001
Clavien Dindo					< 0.001
0	(70.7%)	171	(41.1%)	138	
1–3a	(19.4%)	47	(47.6%)	160	
3b-5	(9.9%)	24	(11.3%)	38	
Reintervention^*^<90 days,					0.571
Yes	(10.3%)	25	(8.9%)	30	
No	(89.7%)	217	(91.1%)	306	
Readmission^*^< 90 days					0.807
Yes	(9.5%)	23	(10.1%)	34	
No	(90.5%)	219	(89.9%)	302	
Mortality					
< 30 days	(1.7%)	4	(0.8%)	3	0.261
30 > days < 90	(0.4%)	1	(1.7%)	6	
No	(97.9%)	237	(97.3%)	327	

Length of stay reported in days. *Including scheduled ileostomy reversal for rectum resections

### Secondary outcomes

Fewer patients in ERAS+ developed a postoperative complication according to the Clavien Dindo classification (*p* < 0.001). No difference was found in the number of reinterventions (*p* = 0.571), readmissions (*p* = 0.807) or mortality (*p* = 0.261). These variables indicate the safety of the ERAS+ protocol, as no added risk of morbidity within 90 days was found (Table 2).

A total of 144 (59.5%) patients were able to meet the EDD, 95 (39.3%) patients were admitted longer than their EDD, and 3 patients died during admission (all three due to cardiopulmonary failure), (Table 3). The majority of patients (67.4%) who did not meet the EDD had a medical cause for prolonged stay. Medical causes were a variety of physical complaints (pain, nausea, vomiting, shortness of breath): fever, abnormal blood values, slow recovery and postoperative complications (ileus, postoperative haemorrhage, anastomotic leakage), including reinterventions. Twenty-one patients who were admitted longer, but met the discharge criteria at EDD, were waiting for postdischarge (home) care. Six patients were not confident enough to return home. For four patients, the cause of prolonged admission was due to a preference of being discharged after the weekend.

**Table 3:** Results of implementing EDD

Expected day of discharge met?	ERAS+, *n* (%)
Yes	144 (59.5%)
No	95 (39.3%)
Medical cause	64 (67.4%)
Waiting for care	21 (22.1%)
Insecurity	6 (6.3%)
Weekend	4 (4.2%)
Deceased during admission	3 (1.2%)

### Multivariate linear regression analysis of predictors for length of stay

In order to investigate these predictors of length of stay, we performed a multivariate analysis: multiple variables were added in the regression model to correct for possible confounders. UICC stage, sex, the Charlson Comorbidity Index and the Clavien Dindo classification were added to the type of ERAS protocol (Table 4). The type of ERAS protocol was an independent outcome predictor for length of stay in the univariable analysis (*p* < 0.001). After conducting a multivariate analysis, the ERAS protocol variable kept its predictive value as it was found to be a confounder (*p* = 0.001), with ERAS+ leading to a decrease in LOS. In addition to this, in the regression model, UICC stage (*p* = 0.029), the Clavien Dindo classification (*p* < 0.001) and the Charlson Comorbidity Index (*p* = 0.031) also showed to be important predictors of length of stay.

**Table 4:** Multivariate linear regression analysis of predictors for length of stay

Variables	Coefficient (β)	95% CI	*p*-value
Univariate			
Age	0.029	−0.031/0.065	0.479
Procedure	0.067	−0.222/2.334	0.105
ERAS+ protocol	−0.145	−2.787/-0.787	<0.001
UICC stage	0.150	0.248/0.816	<0.001
Sex	0.089	0.096/2.098	0.032
CDC	0.512	1.707/2.525	<0.001
CCI	0.090	0.026/0.483	0.029
Multivariate			
ERAS+ protocol	−0.123	−2.420/−0.629	0.001
UICC stage	0.081	0.134/2.544	0.029
Sex	0.044	−0.352/1.437	0.234
CDC	0.433	7.007/9.811	<0.001
CCI	0.080	0.070/1.490	0.031

*Procedure* performed surgical technique, laparoscopic or open, *ERAS* variable used to distinguish the used protocol in the two study groups, *UICC stage the Union for International Cancer Control (UICC) TNM Classification of Malignant Tumours, CDC Clavien Dindo classification, CCI Charlson comorbidity index, CI confidence intervals*

## Discussion

This study investigated the use of perioperative education of the expected day of discharge (EDD) within the ERAS program for patients undergoing colorectal cancer surgery in a teaching hospital. The aim of this study was to reduce length of hospital stay (LOS) by engaging patients more actively in the recovery process, by informing both patients and their family members of the EDD on a daily basis, both preoperatively and during hospital stay. This protocol was named ERAS+, as the standard surgical care in this tertiary hospital was according to the current ERAS guidelines [8].

ERAS+ was found to be feasible and resulted in a decreased LOS of 1 day without increased risk of severe postoperative complications, readmissions or mortality.

Though the type of ERAS protocol was found to be a predictor of hospital LOS, other (pre)operative factors such as comorbidity, tumour stage and the occurrence of severe postoperative complications were also found to affect hospital LOS. These findings are easily explainable and similar to previous reports about the beneficial effects of multimodal enhanced recovery programs for both open and laparoscopic colorectal surgeries [6, 19]. Though no significance was found, more patients in the regular ERAS group had a higher UICC stage compared to those in the ERAS+ group (see Table 1). One possible reason for this discrepancy could be attributed to the introduction of the National Population Screening for Colorectal Cancer here in the Netherlands [20]. After the introduction of this systematic population screening program in 2014, the incidence of colorectal cancer has considerably increased reaching a peak in 2016 of about 15,382 patients diagnosed with colorectal cancer. With increasing efficiency of the screening program, the incidence of colorectal cancer slowly declined to about 14,102 patients in 2018 [21]. This trend is a direct cause of early detection of colorectal disease, leading to lower incidence and mortality [22]. This could possibly also explain the difference in the number of patients recruited in 2016 and 2018.

In most studies, LOS is taken as a measure to assess patient recovery, reported as time to achieve complete recovery of a patient. Balvardi et al. conducted an observational validation study, with the aim to assess the difference between time-to-readiness for discharge and actual LOS as a measure for in-hospital recovery after colorectal surgery. In this study, they found that 40% of patients stayed in the hospital, even after discharge criteria were met (readiness for discharge) [15]. The median difference between time-to-readiness and actual hospital LOS was 1 day (IQR, 1–2). Fifty-seven percent of delayed discharge was related to a medical reason, due to insufficient recovery according to the attending surgeon’s judgment.

As previously described [11, 12], psychological factors seem to play a role in hospital LOS. We reported that 39.3% of ERAS+ patients did not meet the EDD, of which the majority (67.4%) was due to a medical reason. The remaining 32.6% of patients did not meet the EDD due to various psychological and logistical factors. Unfortunately, three patients (1.2%) died during admission (all three due to cardiopulmonary failure). In current practice, logistical (discharge) processes such as home care, family care, rehabilitation and nursing homes interfere and lead to longer hospital LOS. If such logistical factors could be tackled and arranged with and/or for the patient before a surgical procedure, the rate of non-medical prolonged admission could be reduced [23].

Patient education and expectation management actively involve the patient in the surgical recovery process. Lee et al. found that preoperative patient education and expectation management increased patient compliance, as this modified subjective factors related to functional recovery [24]. Factors such as regained mobility, oral intake and bowel movements, were found to be the most important factors influencing a patient’s decision to go home after colorectal surgery. Forsmo et al. found similar results after conducting a randomized controlled trial (RCT) (*n* = 164) assessing the impact of extended perioperative counselling in an ERAS setting [25]. They showed a median difference in length of stay of two days in favour of the group with extended perioperative counselling. They also concluded that adherence to all perioperative ERAS elements in both study groups is crucial.

In the current study, fewer patients in the ERAS+ group developed postoperative complications. More patients developed minor complications (Clavien Dindo grades I–IIIa) in the ERAS group compared to the ERAS+ group (ERAS 160 (47.6%) vs ERAS+ 47 (19.4%), *p* < 0.001. After a closer look, only three patients in the ERAS+ group had a grade IIIa complication and 11 patients in the ERAS group who had a grade IIIa complication. The remaining patients had either a grade I or II complication which includes any deviation from the normal postoperative course with- or without the need for pharmacological treatment. As for the difference in major complications in this current study, ERAS+ 24 (9.9%) vs ERAS 38 (11.3%), no clear reason can be given. However, due to the retrospective nature of this study, sampling bias may have played a role. Interestingly, these findings are similar to those of Forsmo et al., who showed a near significant difference in major complications in the extended counselling group 3 (3.8%) compared to 10 (11.9%) patients in the standard counselling group (*p* = 0.053) [25]. Furthermore, several studies have found an association between (severe) postoperative complication and low patient adherence to ERAS protocols [26–29]. One could suggest that the higher postoperative complication rate in the ERAS group might reflect a possibly lower compliance to the ERAS protocol. Patients included in the ERAS+ group may have had more active monitoring when reminded of their EDD. No other changes or improvements to the standard ERAS protocol were made other than the introduction (and daily information) of the EDD. Because of the retrospective nature of this study, it was not possible to check compliance for each ERAS element of each patient. It remains unclear why the complication rate differed between groups.

Though showing promising results, this current study has its limitations. Its retrospective design may have led to information bias, as the collected information relied on (quality of) the reports in the patients’ electronic file. Also, patients included in the ERAS+ group were on average about 2 years older than patients in the regular ERAS group. Though a statistical significance was found, the clinical relevance and impact hereof are negligible. This also applies to the difference in male-female ratio between the two groups. There were more males in the ERAS group; however, sex was not found to be a significant confounder in de multivariate regression analysis. The clinical relevance (and impact) hereof is negligible.

In conclusion, perioperative education of the expected day of discharge within the ERAS program can reduce length of hospital stay after colorectal surgery. Protocol (ERAS) compliance and expectation management are key factors. This free-of-charge ERAS program ‘extension’ ensures better understanding, faster discharge and lower costs for the hospital without increased risk of rehospitalisation or complications.

## Data Availability

Not applicable.
